# Cleavage site and Ectodomain of HA2 sub-unit sequence of three equine influenza virus isolated in Morocco

**DOI:** 10.1186/1756-0500-7-448

**Published:** 2014-07-12

**Authors:** Mohamed Boukharta, Fathiah Zakham, Nadia Touil, Mehdi Elharrak, Moulay Mustapha Ennaji

**Affiliations:** 1 University Hassan II, Faculty of Sciences and Techniques, Mohammedia-Casablanca, Laboratory of Virology, Microbiology and Quality/ETB, Mohammedia BP 146, (20650), Morocco; 2 Department of Biology, Instruction Military Hospital Med V Rabat, University Mohammed V Souissi, Rabat, Morocco; 3 Société de Produits biologiques et pharmaceutiques vétérinaires (Biopharma), Rabat, Morocco

**Keywords:** Equine influenza, Hemagglutinin (HA), HA2 sequences, Ectodomain (ED), Cleavage site

## Abstract

**Background:**

The equine influenza (EI) is an infectious and contagious disease of the upper respiratory tract of horses. Two outbreaks were notified in Morocco during 1997 and 2004 respectively in Nador and Essaouira. The aims of the present study concern the amino acids sequences comparison with reference strain A/equine/Miami/1963(H3N8) of the HA2 subunit including the cleavage site of three equine influenza viruses (H3N8) isolated in Morocco: A/equine/Nador/1/1997(H3N8), A/equine/Essaouira/2/2004 (H3N8) and A/equine/Essaouira/3/2004 (H3N8).

**Results:**

The obtained results demonstrated that the substitutions were located at Ectodomain (ED) and transmembrane domain (TD), and they have only one arginine in cleavage site (HA1-PEKQI-R^329^-GI-HA2). In the Ectodomain, the mutation N/154^**2**^/T deleted the NGT glycosylation site at position 154 for both strains A/equine/Essaouira/2/2004(H3N8) and A/equine/Essaouira/3/2004(H3N8). Except for mutation D/160^2^/Y of the A/equine/Nador/1/1997(H3N8) strain, the other mutations were involved in non conserved sites. While the transmembrane domain (TM) of the strain A/equine/Essaouira/3/2004(H3N8) exhibits a substitution at residue C/199^**2**^/F. For the A/equine/Nador/1/1997(H3N8) strain the HA2 shows a mutation at residue M/207^**2**^/L. Three Moroccan strains reveals a common substitution at the residue E/211^**2**^/Q located between transmembrane domain TM and the cytoplasmic domain (CD).

**Conclusion:**

The given nature virulence of three Moroccan strains, the identified and reported mutations certainly played a permissive role of infection viral process.

## Background

In Morocco horses play a significant socio-economic role, but their health status is threatened by the occurrence of many infectious and contagious diseases such as equine influenza (EI), equine viral arteritis, rhino-pneumonia and West Nile disease. However, EI remains the most alarming disease that causes significant economic losses, often due to the unavailability of sportive horses and the decline of their performance during pulmonary sequels [1-3].

Equine influenza virus is a species-type A influenza virus from the orthomyxovirus family [4]. Two subtypes of equine influenza virus H7N7 and H3N8, were identified: (prototypes A/equine/Prague/1/56(H7N7) and A/equine/Miami/1/63(H3N8) [5,6]. The first subtype (H7N7) is antigenically stable. Since 1979, no outbreaks have been reported [7,8]. While, the second subtype (H3N8) continues to circulate worldwide, and was responsible of all recent reported outbreaks [9,10]. Since 1989, this virus has diverged into two antigenically distinct lineages: Eurasian and American lineages. The last lineage subsequently diverged into three sublineages: south America, Florida (Clades 1 and 2) and Kentucky [4].

The genome of equine influenza virus (EIV) consists in of eight RNA segments encoding 11 proteins: polymerase polypeptides: PB1, PA, PB2, surface glycoproteins hemagglutinin (HA) and neuraminidase (NA), major nucleocapsid protein (NP), matrix protein (M1), ionic channel protein (M2), nonstructural anti-interferon protein (NS1), nuclear export protein (NEP), and some strains of EIV also express a recently discovered PB1-F2 mitochondrial protein (PB1-F2) [11-13], which is a pro-apoptotic peptide with a predominantly mitochondrial localization.

The hemagglutinin is a glycoprotein encoded by segment 4 of the viral genome and synthesized as a precursor form (HA0) (75 kDa) of 550 amino acids by the ribosomes associated with endoplasmic reticulum [14].

The association of three monomers (HA0) forms a homo-trimer projected form spicules on the surface of the viral particle of 135 A [15]. It consists in two polypeptides, HA1 of 328 amino acids (50 kDa) and HA2 of 221 amino acids (25KDa), linked to each other by a disulfide bond between residues 14 of the HA1 and 137 of the HA2 [16,17]. The cleavage of HA0 occurs at arginine conserved residue 329 of the cleavage site, which includes 19 residues (323–328 of HA1, 329 and 1–12 of HA2 [18].

In the low virulent strains limited to the respiratory tract, this site is usually a unique arginine (eg: HA1-PEKQI-R^329^-GI-HA2 of the strain A/equine/Miami/1963 (H3N8)/AAA43164). In contrast with the highly pathogenic avian strains, this cleavage site consists in several basic residues, forming a consensus sequence type R-X-K/R-R. (eg: HA1-PQRERRRKKR^329^-GL-HA2 of the strain A/Chicken/Hong Kong/258/97(H5N1)/AAC14418). In the cleavage site of highly pathogenic avian influenza viruses (HPAIV), the “RRRKKR” sequence represents the pathogenic cleavage site motif [19].

Habitually, different proteases recognize monobasic sequences and cleave HA0 into HA1 and HA2 [20]. One of them is the tryptase Clara (extracellular protease). Though the HAs in the multibasic site would be cleaved by ubiquitous proteases such as furin (intracellular protease present in the Golgi apparatus), which leads to a much broader tropism of the virus leading to severe systemic infection [21-24].

The HA2 exhibits a relatively high degree of conservation [25,26] and forms the basis of the structural-functional fusogenic activity, which is often considered as one of the essential biological properties of viral infection [27]. Residues 1–185 are the ectodomain (ED) within the N-terminal extremity of 22 amino acid representing the “fusion peptide (PF)”[28], the residues 185–211 are the transmembrane domain (TM) and finally residues 211–221 form the cytoplasmic domain (CD) embedded within the viral particle [17,29].

Through this paper, we will develop the study of the amino acid sequences of the HA2 sub-unit and cleavage site of three strains isolated in Morocco: A/equine/Nador/1/1997(H3N8), A/equine/Essaouira/2/2004(H3N8), A/equine/Essaouira/3/2004(H3N8) [30] and their comparison with those of the equine influenza strains available in the Genbank database, including strains recently isolated in Algeria by Laabassi et al., [31]. Results released concerning the equine influenza strains isolated in the Arabian Maghreb are likely on the HA1 subunit and more particularly on the antigenic sites [30,31].

## Methods

### Viruses

A/equine/Nador/1/97(H3N8) was isolated in Nador from a mule using 11-day-old specific-pathogen-free chicken eggs as described by Kissi [32]. A/equine/Essaouira/2/2004(H3N8) and A/equine/Essaouira/3/2004(H3N8) were isolated from diseased donkey and a horse, respectively, during 2004 outbreaks in Essaouira. Comparing to the epizootic of 1997, the epizootic of 2004 was notified by the international organization of epizootics [33].

The isolates from Essaouira outbreaks were passaged on Madin-Darby canine kidney (MDCK) cells at 34°C in an atmosphere of 5% CO2 in Eagle’s minimum essential medium supplemented with 5% FCS [34].

### Viral RNA extraction and amplification

Viral RNA was directly extracted from isolates using a Purlink viral RNA/DNA-Minikit (Invitrogen, UK) following the manufacturer’s recommended protocol. PCR was performed by Platinum® PCR SuperMix High Fidelity kit (Invitrogen, UK) on cDNA obtained using primers specific for HA1F (CAGGGGATATTTCTGTCAATCATG) HA1R (GCTGCTTGAGTGCTCTTTAGATC), HA2F (ATTACACCAAATGGAAGCATC) and HA2R (AGTAGAAACAAGGGTGTTTTTAAC) at a final concentration of 0.5 μM for primers. Primer design is detailed by Tissier *et* al. [35]. The thermal cycle was programmed as followed: an incubation at 95°C for 2 min, and then 35 cycles of denaturation at 95°C for 30s, 52°C for 1 min for hybridization of HA1 and 48°C for 1 min for HA2 primer hybridization, 72°C for 30s.

### Sequencing HA genes

The amplified HA (HA1 and HA2) products were sequenced. Briefly, the PCR products were purified using EXOSAP-IT (USB) and bidirectionally sequenced by using ABI BigDye1 Terminator v3.1 (Applied Biosystems) on an ABI 3130xl sequencer (Applied Biosystems). Analysis of the produced electrophoregramm was carried out with the sequencing Analysis Software version 5.3.1 (Applied Biosystems). The HA gene in its entirety was sequenced, HA1 and HA2 two polypeptides, which are representing the two subunits of the protein. Subsequently, these sequences were assembled to reconstruct the entire HA; for this purpose, the primers were selected and the two segments overlapped [35].

### Phylogenetic analysis and sequences alignment

We performed phylogenetic analysis of 39 equine influenza strains (including Moroccan and Algerian isolates) published in GenBank database, selected using the neighbor-joining method [36], in which the A/equine/Miami/63 HA sequence was the root. The tree was visualized using MEGA5.1 software (http://megasoftware.net/) [37].

Multiple alignments of the amino acid sequence of HA2 of Moroccan strains were used by Basic Local Alignment Search Tool (BLAST) and MEGA5.1 software.

## Results and discussion

The nucleotide sequences of hemagglutinin of the three Moroccan isolates: A/equine/Nador/1/1997(H3N8), A/equine/Essaouira/2/2004(H3N8) and A/equine/Essaouira/3/2004(H3N8) are recorded in “GenBank” database under the following accession numbers: JQ955607, JQ955609 and JQ955612.

Between 1972 and 2011, fourteen equine influenza strains have been isolated in the Maghrebian Arab region. Their phylogenetic analyses showed their relationships in the various stages of antigenic evolution (Predivergence, European and American lineages) [30,31].

**Figure 1:**
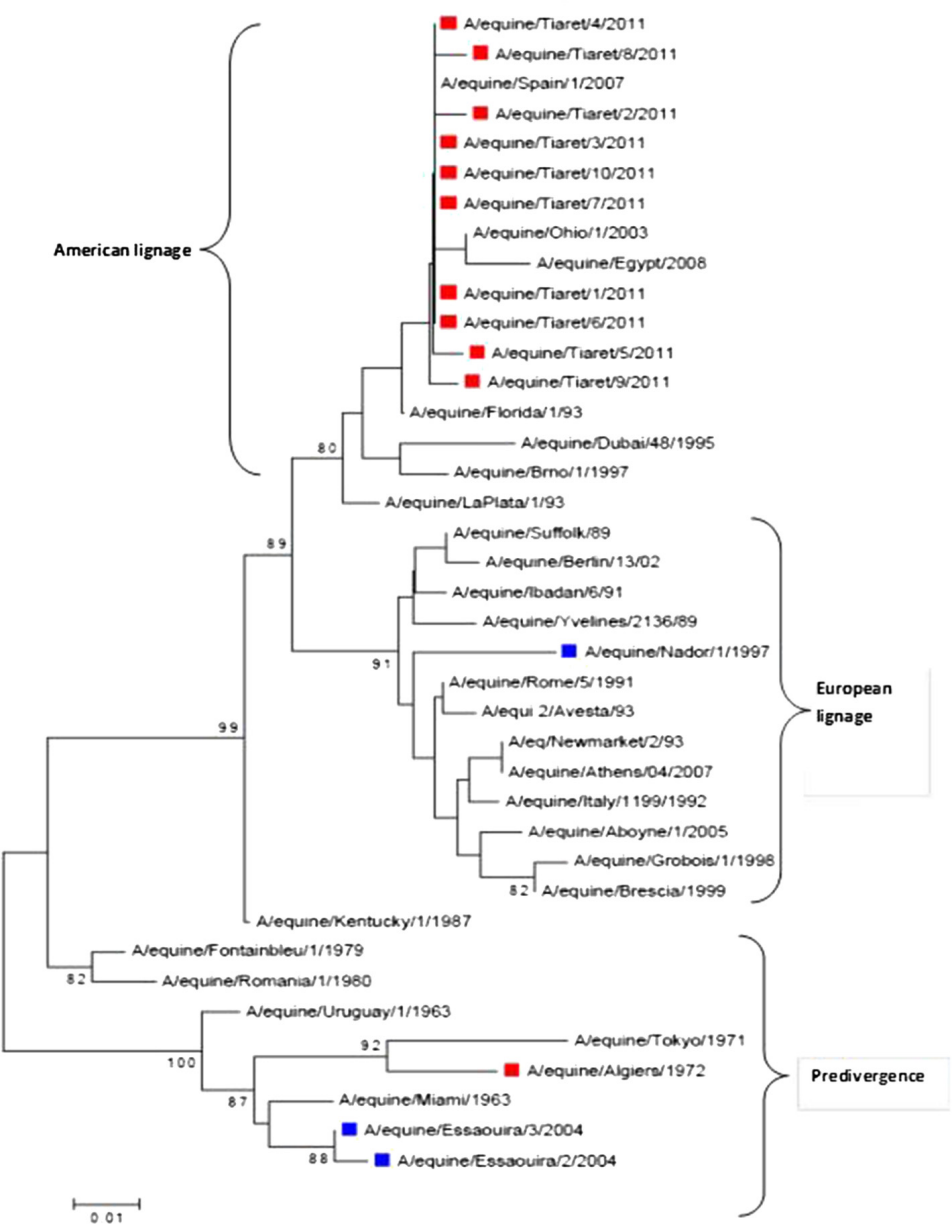
**Phylogenetic tree obtained by comparing amino acid sequences for 30 strains derived from the hemagglutinin gene HA1 (1–329 aa).** Accession numbers: A/equine/Miami/1963, AAA43164; A/equine/Uruguay/1/1963, AAA43114 ; A/equine/Tokyo/1971, AAA43111; A/equine/Algiers/1972 , ACF22126; A/equine/Fontainbleu/1/1979, ACD85396; A/equine/Romania/1/1980, ACD85374; A/equine/Kentucky/1/1987, ACA24568;A/equine/Suffolk/89, CAA48482; A/equine/Yvelines/2136/89 ,BAA33940 ; A/equine/Ibadan/6/91, CAA64893; A/equine/Rome/5/1991, ACD85341; A/equine/Italy/1199/1992 ,ACD85308; A/equine/Florida/1/93, AAB36978; A/equine/LaPlata/1/93,BAA33947; A/equi 2/Avesta/93, CAA74385 ; A/eq/Newmarket/2/93, CAA59416; A/equine/Dubai/48/1995, AEI26218; A/equine/Nador/1/1997, AFJ69903; A/equine/Grobois/1/1998, ACH95594; A/equine/Ohio/1/2003, ABA39846 ; A/equine/Essaouira/3/2004, AFJ69909; A/equine/Spain/1/2007, ADO78886; A/equine/Egypt/6066NAMRU3-VSVRI/2008 (A/equine/Egypt/2008), ACH95682; A/equine/Essaouira/2/2004 ,AFJ69905; A/equine/Athens/04/2007, ADF55752; A/equine/Berlin/13/02, ABP35588 ; equine/Brescia/1999 ,ABU46321 ; A/equine/Brno/1/1997 , AEI26221; A/equine/South Africa/4/2003, ADB45165; A/equine/Richmond/1/2007, ACH95569; A/equine/Tiaret/1/2011, AGR54591; A/equine/Tiaret/2/2011, AGR54592; A/equine/Tiaret/3/2011, AGR54593; A/equine/Tiaret/4/2011, AGR54594; A/equine/Tiaret/5/2011, AGR54595; A/equine/Tiaret/6/2011, AGR54596 ;A/equine/Tiaret/7/2011, AGR54597 ; A/equine/Tiaret/8/2011 ,AGR54598; A/equine/Tiaret/9/2011, AGR54599; A/equine/Tiaret/10/2011, AGR54600.

The strains A/equine/Essaouira/2/2004, A/equine/Essaouira/3/2004 and A/equine/Algiers/1972 show a high homology with reference strain (A/equine/Miami/63) belonging therefore to the predivergence phase (Figure 1). While A/equine/Nador/1/1997 strain was clustered with the Eurasian lineage viruses, exhibiting the highest homology to the influenza viruses in equine infected in Italy in the early nineties (i.e. A/equine/Italy/1199/1992) [30].

In 2011, ten strains of equine influenza (A/equine/Tiaret/1/ 2011 to A/equine/Tiaret/10/2011) were isolated by Laabassi and colleagues in Tiaret (the west of Algeria). These strains belong to the American lineage and Florida sublignage (clade 2) [31] (Figure 1).

The comparison of the amino acid sequences of the HA2 of three strains isolated in Morocco and the reference strain A/equine/Miami/1963(H3N8) shows that the strain A/equine/Nador/1/1997(H3N8) present ten substitutions at the residues respectively: F/56^**2**^/I, K/58^**2**^/R, E/85^**2**^/D, N/135^**2**^/G, E/150^**2**^/G, D/160^**2**^/Y, R/174^**2**^/K, G/175^**2**^/S, M/207^**2**^/L and E/211^**2**^/Q. In both strains A/equine/Essaouira/2/2004(H3N8) and A/equine/Essaouira/3/2004(H3N8), the HA2 represents four common mutations at the residues: F/56^**2**^/I, N/154^**2**^/T, D/158^**2**^/N and E/211^**2**^/Q and an additional substitution at residue C/199^**2**^/F for the strain A/equine/Essaouira/3/2004(H3N8) (Table 1). The strain A/equine/Algiers/1972 has eight substitutions at the residues: Q/327^1^/R, I/328^1^/L, Y/26^2^/H, F/56^2^/I, A/101^2^/T, K/121^2^/R, G/175^2^/S and E/211^2^/Q.

**Table 1:** Mutations comparison of 21 protein sequences of the Hemagglutinin subunit HA2

	**Cleavage site**					**Ectodomain**																										**TM**			
						**FP**	**Short β sheet**	**Helix A**			**Helix B**				**Helix c Coiled coil**			**Helix D**		**Helix E-H**															

**HA residual**	323	325	326	327	328	347	355	369	372	379	385	386	387	391	414	430	431	450	462	464	465	476	479	483	486	487	489	492	496	503	504	527	528	536	540
**HA2 Residual**	-6	-4	-3	-2	-1	18	26	40	43	50	56	57	58	62	85	101	102	121	133	135	136	147	150	154	157	158	160	163	167	174	175	198	199	207	211
A/equine/Miami/1963	V	E	K	Q	I	V	Y	S	A	G	F	E	K	K	E	A	L	K	M	N	G	A	E	N	Y	D	D	R	L	R	G	I	C	M	E
A/equine/Nador/1/1997	.	.	.	.	.	.	.	.	.	.	I	.	R	.	D	.	.	.	.	G	.	.	G	.	.	.	Y	.	.	K	S	.	.	L	Q
A/equine/Essaouira/2/2004	.	.	.	.	.	.	.	.	.	.	I	.	.	.	.	.	.	.	.	.	.	.	.	T	.	N	.	.	.	.	.	.	.	.	Q
A/equine/Essaouira/3/2004	.	.	.	.	.	.	.	.	.	.	I	.	.	.	.	.	.	.	.	.	.	.	.	T	.	N	.	.	.	.	.	.	F	.	Q
A/equine/Algiers/1/1972	.	.	.	R	L	.	H	.	.	.	I	.	.	.	.	T	.	R	.	.	.	.	.	.	.	.	.	.	.	.	S	.	.	.	Q
A/equine/Tokyo/2/1971	I	G	.	R	L	I	H	.	.	.	I	.	.	.	.	T	.	R	I	.	.	.	.	.	.	.	.	.	.	.	.	.	.	.	Q
A/equine/Uruguay/1/1963	.	.	R	.	.	I	.	.	.	.	I	G	.	.	.	.	.	.	.	.	.	.	.	.	.	.	.	K	.	.	.	.	.	.	Q
A/equine/Sao Paulo/6/1963	.	.	R	R	L	I	.	.	.	.	I	G	.	.	.	.	I	.	.	.	R	T	.	.	.	N	.	.	V	K	.	.	.	.	Q
A/equine/Sachiyama/1/1971	I	.	.	R	L	I	H	.	.	.	I	.	.	.	.	T	.	R	I	.	.	.	.	.	.	.	.	.	.	.	.	.	.	.	Q
A/equine/Italy/1199/1992	.	.	.	.	.	.	.	.	.	.	I	.	R	.	.	.	.	.	.	G	.	.	G	.	.	.	Y	.	.	K	S	.	.	.	Q
A/equine/Egypt/2008	.	.	.	.	.	.	.	.	.	.	I	.	R	.	.	.	.	.	.	G	.	.	G	.	.	.	Y	.	.	K	.	V	.	.	Q
A/equine/Tiaret/1/2011	.	.	.	.	.	.	.	.	T	E	I	.	R	.	.	.	.	.	.	G	.	.	G	.	.	.	Y	.	.	K	.	.	.	.	Q
A/equine/Tiaret/2/2011	.	.	.	.	.	.	.	.	T	E	I	.	R	.	.	.	.	.	.	G	.	.	G	.	.	.	Y	.	.	K	.	.	.	.	Q
A/equine/Tiaret/3/2011	.	.	.	.	.	.	.	.	T	E	I	.	R	.	.	.	.	.	.	G	.	.	G	.	.	.	Y	.	.	K	.	.	.	.	Q
A/equine/Tiaret/4/2011	.	.	.	.	.	.	.	.	T	E	I	.	R	.	.	.	.	.	.	G	.	.	G	.	.	.	Y	.	.	K	.	.	.	.	Q
A/equine/Tiaret/5/2011	.	.	.	.	.	.	.	.	T	E	I	.	R	.	.	.	.	.	.	G	.	.	G	.	.	.	Y	.	.	K	.	.	.	.	Q
A/equine/Tiaret/6/2011	.	.	.	.	.	.	.	.	T	E	I	.	R	.	.	.	.	.	.	G	.	.	G	.	C	.	Y	.	.	K	.	.	.	.	Q
A/equine/Tiaret/7/2011	.	.	.	.	.	.	.	.	T	E	I	.	R	.	.	.	.	.	.	G	.	.	G	.	.	.	Y	.	.	K	.	.	.	.	Q
A/equine/Tiaret/8/2011	.	.	.	.	.	.	.	.	T	E	I	.	R	.	.	.	.	.	.	G	.	.	G	.	.	.	Y	.	.	K	.	.	.	.	Q
A/equine/Tiaret/9/2011	.	.	.	.	.	.	.	.	T	E	I	.	R	.	.	.	.	.	.	G	.	.	G	.	.	.	Y	.	.	K	.	.	.	.	Q
A/equine/Tiaret/10/2011	.	.	.	.	V	.	.	.	T	E	I	.	R	E	.	.	.	.	.	G	.	.	G	.	.	.	Y	.	.	K	.	.	.	.	Q

Accession numbers: A/equine/Miami/1963, AAA43164; A/equine/Nador/1/1997, AFJ69903; A/equine/Essaouira/2/2004, AFJ69905; A/equine/Essaouira/3/2004, AFJ69909; A/equine/Algiers/1/1972, ACF22126; A/equine/Italy/1199/1992, ACD85308; A/equine/Sachiyama/1/1971, ACI25735; A/equine/Sao Paulo/6/1963, ACD85264; A/equine/Egypt/6066NAMRU3-VSVRI/2008 (A/equine/Egypt/2008), ACH95682; A/equine/Uruguay/1/1963, ACD85418; A/equine/Tokyo/2/1971, AEM60147; A/equine/Tiaret/1/2011, AGR54591; A/equine/Tiaret/2/2011, AGR54592; A/equine/Tiaret/3/2011, AGR54593; A/equine/Tiaret/4/2011, AGR54594; A/equine/Tiaret/5/2011, AGR54595; A/equine/Tiaret/6/2011, AGR54596; A/equine/Tiaret/7/2011, AGR54597; A/equine/Tiaret/8/2011, AGR54598; A/equine/Tiaret/9/2011, AGR54599; A/equine/Tiaret/10/2011, AGR54600.

Ten Algerian strains (A/equine/Tiaret/1/2011 to A/equine/Tiaret/10/2011) presented nine common mutations at residus: A/43^**2**^/T, G/50^**2**^/E, F/56^**2**^/I, K/58^**2**^/R, N/135^**2**^/G, E/150^**2**^/G, D/160^**2**^/Y, R/174^**2**^/K, E/211^**2**^/Q. both strains A/equine/Tiaret/10/2011 and A/equine/Tiaret/6/2011 show additional mutation respectively at residue I/328^**1**^/V et K/62^2^/C. The impact of adjacent amino acids of HA cleavage site on virulence was studied by several authors [38].

The hemagglutinin of the three isolated strains in Morocco A/equine/Nador/1/1997, A/equine/Essaouira/2/2004(H3N8) and A/equine/Essaouira/3/2004 (H3N8) have a single conserved arginine at the residue 329 (R329), while the strain A/equine/Algiers/1972 (H3N8) shows two arginines at the residues 327 and 329 (Q/327^**1**^/R, R329) (HA1-RXR^329^-GI-HA2). Mutations leading to the acquisition of basic amino acids are also observed for the strains A/equine/Tokyo/2/1971(H3N8) (Q/327^1^/R, HA1-RXR^329^-GI-HA2), A/equine/Uruguay/1/1963(H3N8) (K/326^1^/R, HA1-RXXR^329^-GI-HA2), A/equine/Sao Paulo/6/1963 (H3N8) (K/326^1^/R et Q/327^1^/R, HA1-RRXR^329^-GI-HA2 and A/equine/Sachiyama/1/1971 (H3N8) (Q/327^1^/R, HA1-RXR^329^-GI-HA2) (Figure 1).

Interestingly, these mutations do not corroborate the consensus sequence of the high pathogenic avian virus H5 and H7 (HA1-RRRKKR^329^- GL-HA2) [24].

**Figure 2:**
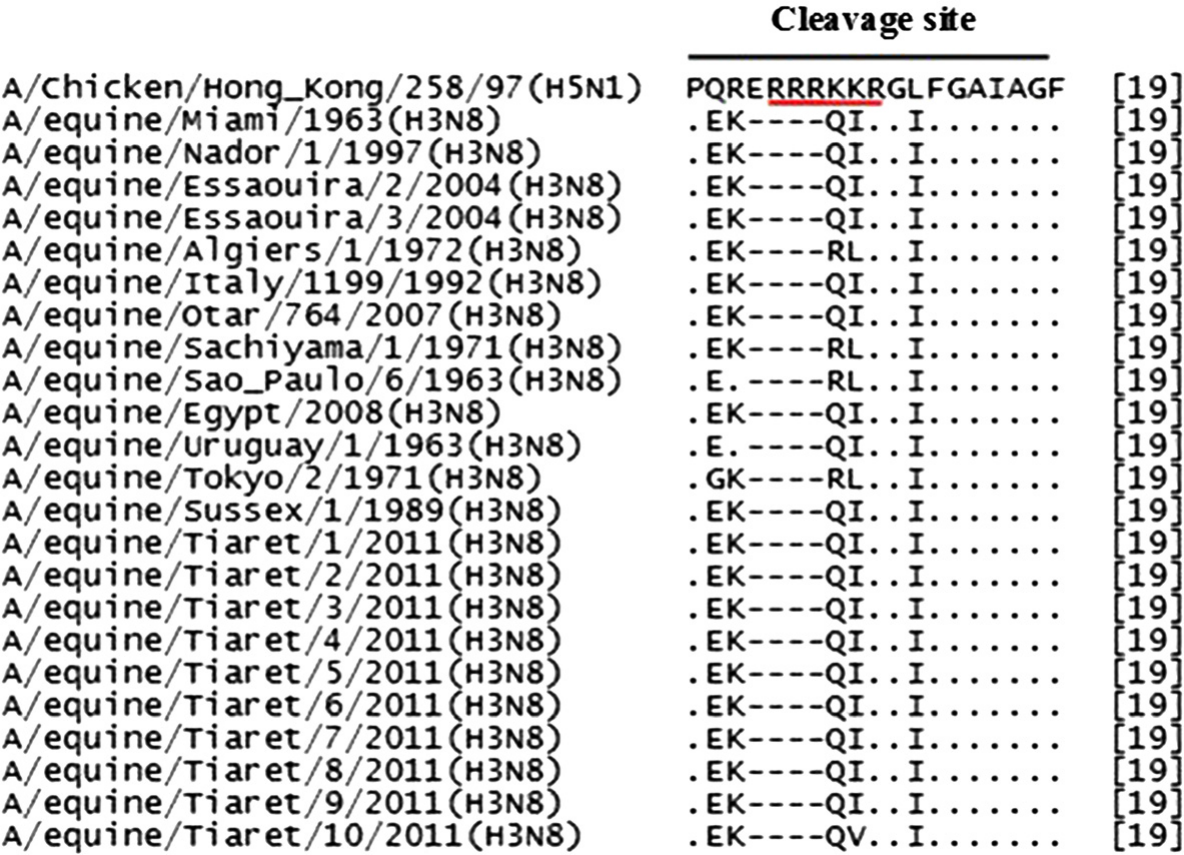
**Multiple alignment of 24 sequences of cleavage site (Hemagglutinin HA2).** (Red line: sequence forming pathogenic cleavage site motif). Accession numbers: A/Chicken/Hong Kong/258/97(H5N1), AAC14418; A/equine/Miami/1963, AAA43164; A/equine/Nador/1/1997, AFJ69903; A/equine/Essaouira/2/2004, AFJ69905; A/equine/Essaouira/3/2004, AFJ69909; A/equine/Algiers/1/1972, ACF22126; A/equine/Italy/1199/1992 , ACD85308; A/equine/Otar/764/2007, ADZ55424; A/equine/Sachiyama/1/1971, ACI25735; A/equine/Sao Paulo/6/1963, ACD85264; A/equine/Egypt/6066NAMRU3-VSVRI/2008 (A/equine/Egypt/2008), ACH95682; A/equine/Uruguay/1/1963, ACD85418; A/equine/Tokyo/2/1971, AEM60147; A/equine/Sussex/1/1989, ACD97425; A/equine/Tiaret/1/2011, AGR54591; A/equine/Tiaret/2/2011, AGR54592; A/equine/Tiaret/3/2011, AGR54593; A/equine/Tiaret/4/2011, AGR54594; A/equine/Tiaret/5/2011, AGR54595; A/equine/Tiaret/6/2011, AGR54596 ;A/equine/Tiaret/7/2011, AGR54597 ; A/equine/Tiaret/8/2011 ,AGR54598; A/equine/Tiaret/9/2011, AGR54599; A/equine/Tiaret/10/2011, AGR54600.

Figure 2 shows a cleavage sites sequences comparison with the highly pathogenic avian strain A/Chicken/Hong Kong/258/97 (H5N1)/AAC14418). It’s worth noting that a pathogenic cleavage site motif “RRRKKR” is absent in the cleavage site of the equine influenza virus (H3N8). However, Bogs et al., 2010, reported that the introduction of such a polybasic motif into the HA cleavage site of a low-pathogenic H3N8 strain did not lead to transformation into an HPAIV indicating the existence of additional virulence determinants in the HA and/or the other viral proteins [39].

Furthermore, the passage of a monobasic cleavage site to another polybasic represents a potential risk of increased virulence and spread of influenza infection [19].

The mutations at the cleavage site by basic amino acids for EIVs are exceptional. The result of the alignment of 132 hemagglutinin sequences (H3) (full length) of EI with available sequences on the Genbank database was made. Only nine sequences (ACF22126, AAA43100, ACI25735, ACD85385, ACD85264, AAA43111, AEM60147, AAA43114, and ACD85418) have at least two arginines at the cleavage site. The three types of sequences HA1-RXR^329^-GI-HA2, HA1-RXXR^329^-GI-HA2 and HA1-RRXR^329^-GI-HA2 are noticed.

Among 23 amino acids “GIFGAIAGFIENGWEGMVDGWYG” corresponding to the sequence of the fusion peptide of the three Moroccan isolated and the used aligned strains, the four residues 20–23 (GWYG) are completely conserved which include two hydrophobic residues of Glycine [23].

Additionally, the result of amino acid alignment shows no mutation compared to the reference strain A/equine/Miami/1963. The high conservation of the N-terminal region of the HA2 sequences, especially for the first 11 amino acids “GIFGAIAGFIE”, with few rare mutations, was observed in all HA subtypes of influenza virus type A. Currently, this conserved region of HA2 and of the M2e (ectodomain M2 ion-channel protein) form the basis of the research for the development of a universal vaccine [40,41].

The three segments (residues 34–37) of the homo-trimer comb the cap of the N-terminal triple-stranded coiled coil forming a small ring. Even more, the annular space formed is stabilized by the triple contact between the methyl groups of Ala-35 and Ala-36, which are highly conserved [17]. At the ectodomain, particularly the long alpha helix composed of 68 residues (38–105), which consists of three helices A (residues 38–55), B (residues 56–75) and C (76–105) [15], the strains A/equine/Algiers/1972(H3N8) A/equine/Essaouira/2/2004(H3N8) and A/equine/Essaouira/3/2004(H3N8) have a common single substitution at residue F/56^2^/I.

Moreover, the strain A/equine/Algiers/1972(H3N8) has a second mutation at residue A/101^2^/T. For the strain A/equine/Nador/1/1997(H3N8), the HA2 has three mutations at the residues F/56^**2**^/I, K/58^**2**^/R, E/85^**2**^/D. At the helix D (106–129), the strain A/equine/Algiers/1972(H3N8) has a single substitution at residue K/121^2^/R. At the helix formed E-H residues (130–175), the strain A/equine/Nador/1/1997(H3N8) presents four mutations at the residues E/150^**2**^/G, D/160^**2**^/Y, R/174^**2**^/K and G/175^**2**^/S.

Considerably, the mutation D/160^2^/Y of the strain A/equine/Nador/1/1997(H3N8) localized on a conserved site, which characterizes the viruses of subtype (H3) [22]. The A/equine/Algiers/1972(H3N8) strain exhibits a substitution at residue G/175^2^/S. Regarding the A/equine/Essaouira/2/2004(H3N8) strain and A/equine/Essaouira/3/2004(H3N8) strain, the HA2 presents two common mutations at the residues N/154^**2**^/T et D/158^**2**^/N. The first mutation (N/154^**2**^/T) affects a glycosylation site (Figure 1). This mutation removed the NGT glycosylation site at position 154, which, could affect the structure, flexibility, and solvent exposure of this region. However, the recent adaptation of equine H3N8 virus to dogs is associated with five HA mutations, which include the N154^2^T [42].

At the transmembrane domain TM of the strain A/equine/Essaouira/3/2004(H3N8) exhibits a substitution at residue C/199^**2**^/F. For the A/equine/Nador/1/1997(H3N8) strain the HA2 shows a mutation at residue M/207^**2**^/L. The four Maghreb strains reveals a common substitution at the residue E/211^**2**^/Q situated between transmembrane domain TM and the cytoplasmic domain (CD).

Indeed, the occurrence of severe respiratory signs in the infected horses, obviously confirm that the enrolled four strains in this study are virulent in nature.

## Conclusion

Although the amino acid sequences of the HA2 subunit are relatively stable, several mutations compared to the reference strain A/equine/Miami/1963 were found for the four strains of the Arabic Maghreb. Apart from the mutation D/160^2^/Y of the strain A/equine/Nador/1/1997(H3N8), Q/327^1^/R of the strain A/equine/Algiers/1972(H3N8) and N/154^2^/T of both strains A/equine/Essaouira/2/2004 (H3N8)and A/equine/Essaouira/3/2004(H3N8), the other mutations were involved at not conserved sites at helices B, C, D, E-H and TM.

## Competing interests

The authors declare that they have no competing interests.

## Authors’ contributions

The work presented here was carried out in collaboration between all authors. BM performed and wrote the first draft of the manuscript. FZ helped in redrafting the manuscript. MME, EHM, TN are conceived of the study and helped revisinging the manuscript. All authors read and approved the final manuscript.
